# Human Factors and Organizational Issues in Health Informatics: Review of Recent Developments and Advances

**DOI:** 10.1055/s-0044-1800744

**Published:** 2025-04-08

**Authors:** Andre Kushniruk, David Kaufman

**Affiliations:** 1 School of Health Information Science, University of Victoria, Victoria, Canada; 2 Health Informatics, School of Health Professions, SUNY Downstate Health Sciences University, Brooklyn, New York

## Abstract

Objective: In this paper we focus on a review of key articles published in the past two years (2022 and 2023) in the areas of human factors and organizational issues in health informatics.

Methods: We reviewed manuscripts that were published in primary human factors, human factors engineering and health informatics journals. This involved conducting a series of searches using PubMed, Web of Science, and Google Scholar for articles related to human factors in healthcare published in 2022 and 2023.

Results: The range of applications that have been designed and analyzed using human factors approaches has been rapidly expanding, including increased number of articles around topics such as the following: AI in healthcare, patient-centered design, usability of mHealth, organizational issues, and work around ensuring system safety. This includes study of applications designed for use by both patients and health providers applying both qualitative and quantitative approaches to user requirements, user-centered system design and human factors analysis and evaluation.

Conclusion: The importance of human factors is becoming recognized as new forms of health technology appear. A multi-level perspective on human factors, that considers human factors at multiple levels, from the individual user to the complex social and organizational context, was described to consider the range and diversity of human factors approaches in healthcare. Such an approach will be needed to drive the design and evaluation of useful and usable healthcare information technologies.

## 1. Introduction

It is increasingly recognized that the design of usable and useful health technologies depends on careful consideration of human factors at multiple levels [ 1 ]. This includes consideration of not only usability and usefulness at the level of the individual user but also consideration of organizational and policy issues and concerns that ultimately affect adoption of the technologies [ 2 3 4 ]. Over the past two years, a wide range of studies have been published in the literature addressing these issues. In this paper we will highlight and review prominent trends that have emerged from the literature over this period. This has included advances in methods for assessing healthcare systems and their fit with users and organizations. This has ranged from work conducted in laboratory settings and simulation laboratories [ 5 ] to evaluation of technology in the real world using naturalistic approaches as well as remote evaluation [ 6 , 7 ].

Overall, the diversity of types of technologies has rapidly expanded, including a rapid increase in the number of applications applying artificial intelligence in healthcare [ 8 9 10 11 ]. In addition, broadened perspectives on the scope of human factors in health to include a perspective that views the need for design and implementation of systems to be in line with a holistic “One Health” view of health that considers the relation among humans, animals and the environment [ 12 , 13 ], a topic which has garnered increasing interest over the past couple of years. We also look at phenomena from a broader systems-centered perspective by focusing on a widely used framework for understanding outcomes within complex socio-technical systems, the Systems Engineering Initiative for Patient Safety (SEIPS) [ 14 ]. At the organizational level, patient safety culture has become a focal point for promoting institutional changes in the promotion of safer practices and in the reduction of harm. The efforts to study patient safety culture are widespread, global and continuing.

Research around deploying and examining the human factors related to mobile and ubiquitous health have also increased [ 15 , 16 ]. In particular, studies focused on design of applications for use by patients and citizens has increased rapidly [ 17 ], while other studies continue to focus on clinical applications designed for use by health professionals [ 18 19 20 ]. This increase in the range of technologies and applications has also necessitated design and application of a wider range of methods for evaluating technologies from a human factors and organizational perspective. This has included applying data science and computational approaches to evaluation as well as application of AI approaches for analyzing human factors data [ 4 ]. Issues related to ensuring the accuracy and safety of healthcare technologies has also continued as a strong focus of study within health informatics [ 21 , 22 ]. We will discuss these advances and trends in several sections to highlight the focus of much of the work that has been conducted over the past two years in the area of human factors and organizational issues.

## 2. Methods

We employed an approach consistent with previous reviews of human and organizational factors that endeavored to strike a balance between breadth and depth. Our methodology involved conducting a series of searches using PubMed, Web of Science, and Google Scholar for articles published in 2022 and 2023. Additionally, we reviewed each volume of the Journal of the American Medical Informatics Association, Applied Clinical Informatics, JMIR Human Factors, and the International Journal of Medical Informatics. Our search strategies included keyword searches, MeSH terms, and the application of filters to narrow the search scope appropriately. Key terms used in our search included “human factors,” “patient safety,” “medication errors,” “usability,” “systems-centered approach,” and “organizational factors.” We also utilized a “pearl-growing strategy” also referred to as a citation mining strategy [ 23 ]. It involves leveraging found articles to find other relevant ones. We first identified relevant articles cited by the journal articles, using the “related articles” function in Google Scholar and the “related records” feature in Web of Science. PubMed offers a range of functions to support pearl growing including “similar articles”, “cited by” and MeSH terms employed in the article. While our searches aimed to cover as much ground as possible, achieving exhaustive coverage was not feasible. The body of literature on human factors in healthcare is voluminous. A search of articles with the key term “human factors” with the search restricted to the years 2022 and 2023 yielded 65,710 results. A Boolean search including both human factors and health informatics yielded 1,605 results.

We selectively included articles that aligned with the themes we were addressing which focused on information technologies and organizational factors. We endeavored to strike a balance between brevity and informativeness in our presentation. For example, the articles centered around the use of technologies could be presented more concisely because we may presume that the reader has enough background knowledge. On the other hand, those related to the culture of patient safety necessitated the inclusion of greater context and more expansive descriptions. The vast majority of articles included in this review are journal articles and mostly original empirical studies. However, we also included systematic and other reviews, position papers and theoretical frameworks. The review also included book chapters and technical reports.

## 3. Results

### 3.1. Human Factors of Artificial Intelligence and Emerging Technologies

The range of technologies being studied from a human factors and organizational perspective has grown rapidly over the past two years. Many of these studies have focused on human factors and organizational issues in the context of the deployment of increasingly advanced technologies. As the field moves from demonstration and prototype systems in areas such as artificial intelligence to real world applications, the user experience (UX) with these technologies has begun to gain considerable interest [ 24 25 26 ]. While many studies continue to focus on areas related to use and usability of electronic health record systems [ 18 , 20 , 27 28 29 ] the range of related technologies has expanded to include other applications, including many systems that interface with electronic health records (EHRs), such as medication management systems [ 30 31 32 ]. These include applications focused on customization and integration of systems in clinical settings, including the human factors related to increased interoperability and the greater access to health data by both health providers and patients using technologies such as patient portals and virtual care applications [ 26 , 33 ]. The focus of studies of human-computer (HCI) interaction with this increasing and diverse range of technologies characterized many of the studies with a focus on user experience with the technology, as well as a continued interest in their accuracy and safety [ 21 ]. Integration of these new technologies into existing healthcare workflow has continued to be the focus of a number of studies taking a human factors perspective on implementation [ 34 ].

A wide and ever-increasing range of personal health and medical devices continue to be reported. This has included studies of use of smart phone, smart watches, wearable devices and other mobile (mHealth) applications [ 35 36 37 ]. The widespread use of technology by the general population has fueled studies of these devices and their capabilities, including monitoring and alerting functionality. Concerns about both the usability and the safety of such technology has motivated a number of studies focused around human factors issues related to ethics, legality and the role of human-computer interaction (HCI) [ 38 , 39 ]. The Internet of Things (IoT) and the usability and acceptance of IoT based tools and devices was another area of interest [ 40 ]. This included issues around human factors being a potentially weak point leading to possible data breach incidents involving IoT, as well as human factors issues around lay person interaction with these devices, which will be continuing issues into the future as this technology becomes more pervasive.

A rapidly increasing number of articles appeared focusing on human factors articles related to artificial intelligence (AI) applications in healthcare, as this has become a major trend that will continue into the future [ 4 , 11 , 41 ]. Some of the studies of AI application have reported on the barriers and facilitators of adoption of AI technology (from multiple perspectives, from the level of usability studies to research focused at the organizational level), while others reported on a range of human factors related to ethical and legal concerns [ 3 , 21 , 42 ]. A range of studies of the impact of generative AI have also appeared in the past two years, with some focusing on the usefulness and usability of Large Language Model (LLM) applications [ 43 ]. Some human factors studies have focused on clinical applications of AI [ 44 ], while other studies have focused on evaluation of these emerging technologies from an organizational context [ 2 ]. This also included reported work on human-robot interaction in healthcare, with an increasing number of studies examining human-robotic interaction in healthcare contexts and settings [ 45 , 46 ]. In addition, the use of social robots has garnered considerable interest to researchers in both human factors and robotic engineering [ 47 ]. Such work has identified that aspects of human factors related to understanding how to develop empathetic interactions between robot and humans will be key to their adoption and acceptability in healthcare settings. Applications ranging from application of robots for use in areas such as dementia care, assistive living and monitoring of patient show considerable promise.

Development of new ways of visualizing data, in conjunction with methods for analyzing and understanding big data, has also been the focus of an increasing number of publications, including work on design and evaluation of dashboards from a human factors perspective [ 48 ]. Such visualization work can include application of artificial intelligence and data science methods, along with simulation approaches to display information in healthcare contexts in new and more effective ways, including using augmented reality. The design and evaluation of visualization techniques have been reported focusing on human understanding and cognition in responding to innovative display modes, and mechanisms [ 27 ]. This has lead to advances in many areas of healthcare ranging from radiology and pathology to analysis of large data sets used in applications ranging from genomics to predictive analytics. In 2022 and 2023 a growing body of articles began to appear focusing on human factors aspects of generative AI and Chat GPT, with implications for a range of application in healthcare. This has included studies of the accuracy and impact of medical advice provided by such AI applications, as well as their integration into healthcare workflow and practices, which will continue to be an areas of increased focus [ 43 , 49 , 50 ]. The application of virtual reality and understanding its usability and adoption from a human factors perspective was another area of work, including usability studies of virtual and augmented reality applications [ 51 52 53 54 55 56 ]. The range of applications includes using such technology for patient education as well as application in areas including planning for healthcare procedures, operations and conducting realistic simulations of complex healthcare processes.

### 3.2. The Human Factors of Patient-facing Technologies

Over the past couple of years, a growing range of studies focused on design, development and evaluation of novel technologies for patients and lay people [ 57 58 59 ]. Studies focused on the remote interaction among patients and healthcare providers using information technology has formed another trend along these lines [ 60 ]. This has included studies of initiatives such as hospital at home and remote patient monitoring, which have been evaluated from a human factors perspective [ 61 ]. Other technologies such as wearable technologies and advances in mHealth applications have been the focus of testing in a number of studies to establish the feasibility of employing them in real world settings and moving the technology from the lab to general use [ 16 , 62 ]. As reported in a number of studies much of this work has focused on assistive living and technology designed to allow the elderly and disabled to stay at home, as well as applications for dementia and other patients [ 63 ]. Most studies have focused on understanding the relation between patients and remote monitoring devices [ 64 65 66 ]. This included exploration of human factors issues around trust, privacy and confidentiality of data collected from remote monitoring.

e-Health literacy is another area of increased focus in the literature, with a range of technologies being developed with the objective of allowing patients to be able to effectively use personal health apps, patient portals to interact with health systems, web sites and informational resources directly [ 67 68 69 70 ]. Since the COVID epidemic there has been an increase in work from a human factors perspective in providing effective virtual care and supporting patient interaction with their providers and the overall health system [ 71 72 73 ]. To analyze the patient experience, studies employing tools such as patient journey mapping, process modelling and analysis of care pathways have also been reported [ 74 ]. Identification of areas within the patient journey where information technology could be designed to interact with both patients and providers is a promising line of research that lead to a patient-centric approach to health informatics [ 71 ].

### 3.3. The EHR and Beyond: Human Factors in Clinical Informatics

Several studies continue to focus on clinician ( *e.g.* , physician or nurse) interaction with the electronic health record (EHR) [ 29 , 75 , 76 ]. Many of these studies addressed clinical decision support and the usability of these systems has continued to be a focus of much study, as reports of poor usability and human factors concerns persist. Ethnographic studies of the use, adoption and customization of EHR technology has included work on relating electronic health record issues and diagnostic error [ 28 ]. Others have reported on work in developing parallel methods (including usability inspection, interviews and questionnaires) for analyzing and improving the usability of computerized decision support for pharmacy and related clinical systems [ 77 ]. It is expected that work in this area of improving the usability of EHRs will continue into the future, employing both small scale usability studies as well as larger scale quantitative analyses of user interactions [ 76 ].

### 3.4. Advances in Models and Methods

Over the past two years, a wide range of methodological approaches have been employed in studies in human factors. In addition, a number of papers continue to report on the development of new or novel approaches to designing and evaluating healthcare technology. This includes articles on user-centered design as well as participatory design methods [ 78 , 79 ] and mixed method approaches [ 80 , 81 ]. Methodologies reported have ranged from laboratory-based studies to testing of systems in real world or simulated settings, with an increased focus on agile approaches [ 82 83 84 ]. Advances in methods for conducting usability testing as well as inspection methods were also reported [ 85 ]. For the requirements gathering and design phases of the System Development Life Cycle (SDLC), a range of approaches to both eliciting user needs and developing rapid prototypes have been reported. Understanding user needs formed the focus of a number of studies and the use of methods such as focus groups, contextual inquiry and ethnographic approaches have been reported [ 58 , 67 ]. This included approaches inspired by models of socio-technical design [ 86 ].

Advances in the measurement of user perceptions and use of validated scales have continued to appear in numerous studies. Instruments such as the SUS (System Usability Scale) [ 48 , 87 , 88 ], TAM (Technology Acceptance Model) [ 32 , 40 , 52 ], UTAUT (Unified Theory of Acceptance and Use of Technology) [ 50 , 69 , 89 90 91 92 ], and SUMI (Software Usability Measurement Inventory) continued to be applied in a large number of studies [ 93 ]. Models and frameworks from distributed cognition [ 94 ] also appeared, particularly in studies that have moved from the laboratory to the real world settings, continuing an ongoing trend [ 95 , 96 ]. Socio-technical frameworks and models also continue to be applied for understanding complex interactions in real-world contexts and applications of healthcare technology from both a technical and social lens [ 22 ].

Participatory design and evaluation methods were employed in a wide range of studies and the co-creation of digital health solutions was a popular topic [ 46 , 97 ]. New approaches to patient-centered design of healthcare systems have emerged and were described in a number of papers [ 98 ]. Some of this research argued for a patient-centered approach that puts the patient at the centre of design and evaluation processes. This is a trend that will likely accelerate into the future as it supports a general movement towards patient empowerment, whereby patients become more involved in their own healthcare decision making.

Physician and other health professional burn-out and stress related to use of technology has appeared as an issue that has been a focus of an increasing number of studies [ 99 ]. Some of these studies have employed methods for assessing cognitive load associated with the introduction of technologies [ 100 ], such as the NASA TLX (Task Load Index), as well as use of physiological monitoring [ 101 ]. The study of the impact as well as reduction of documentation burden through application of technology such as AI has also garnered increased interest [ 102 ].

As described in a number of studies there has been a move from the study of HCI exclusively in lab settings to evaluation of systems in real world settings and in-situ testing of applications. The automated collection and analysis of usability testing data, as well as other forms of data emerging from human factors studies, also emerged as a trend in the literature. As qualitative and mixed methods studies of usability continue in the literature a move towards quantifying the user experience has also emerged [ 103 ]. Large scale usability studies, involving collection of large amounts of user tracking and experience data continue to complement smaller scale, more in-depth usability studies, typically focused on in-depth study of cognitive aspects of technology and their impact on human decision making and reasoning [ 76 ].

### 3.5. Virtual Care and Telehealth

In the aftermath of the COVID-19 pandemic a growing number of studies have focused on the move towards virtual healthcare [ 104 ]. Assistive living devices that can be deployed in the home were the focus of a range of studies, often with a focus on how to integrate the devices into the home, their usability and issues related to training of end users, including the elderly [ 105 ]. Many studies, particularly from the computer science and engineering literature, have focused on study of particular devices and technologies somewhat in isolation of their projected use ( *e.g.* , in the home) [ 106 ]. However, researchers in healthcare are beginning to include greater focus on aspects of human factors that are critical to consider in order to move from isolated testing of devices to understanding the complex factors at play when attempting to deploy virtual care to the patient and general population [ 107 ]. Some of this research has begun to delve into the experience of underserved populations with virtual healthcare [ 108 ]. Research into the human factors of telehealth was also an area of growing interest [ 109 ], including a focus on telehealth in primary care [ 110 ].

### 3.6. Multi-Modal User Interaction in Healthcare

Over the past couple of years there has been an increase in the number of studies focused on multi-modal applications [ 111 ]. Such application incorporate multiple ways for supporting human interaction with systems [ 112 ]. Some of the articles along these lines focused on spatial computing, visual computing, haptic interfaces, augmented reality as well as rapid advances in natural language interfaces [ 113 ]. This will be an area that will likely continue to be of importance and will expand as these technologies become used more widely. This includes new approaches to providing context sensitive delivery of information dependent on the context of use of technology, with switching between different user interaction modes, including physical interaction, verbal interaction and non-verbal interaction, being explored [ 111 ] particularly in the space of human-robot interaction in healthcare.

### 3.7. Safety and Technology-Induced Error

Integrally related to the usability of healthcare applications and systems is the study of system and device safety. This has traditionally included studies showing the benefits of application of technology to improve patient and medical safety. However, a growing number of publications have focused on how such technology may inadvertently decrease safety and potentially lead to error [ 71 ]. Technology-induced error can result from the application of technology under real world conditions of stress and urgency and may appear even in systems that have otherwise been deemed to be safe using conventional testing methods [ 21 ]. Detecting and rectifying such error requires application of approaches ranging from simulations to chart audits and retrospective analysis of errors and adverse events [ 114 ]. A number of these studies have now moved from demonstrating this effect to mapping out the frequency of technology-induced error, developing news ways of tracking and quantifying such error, as well as developing human factors methods and approaches aimed at mitigating technology-induced error, ensuring the safety of new technology in healthcare and optimizing the potential benefit of healthcare technology for improving the safety and efficiency of healthcare processes [ 22 ]. It has been argued that an approach to testing safety-critical application is needed in healthcare that could involve applying a set of complementary methods sequentially, for example usability inspection followed by usability testing and naturalistic evaluation in order to catch potential errors more effectively [ 21 ]. This is an important trend that will likely continue as more complex applications appear requiring more extensive user testing.

### 3.8. A System-Centered and Organizational Approach

Human factors approaches differ considerably in the scope of their analysis [ 115 ]. The Institute of Medicine's (IOM) report To Err is Human: Building a Safer Health System [ 116 ], highlighted the significant number of preventable deaths caused by medical errors in the United States, emphasizing that most of these errors are due to systemic issues rather than individual failures of healthcare providers (IOM). The report brought healthcare problems to the attention of the human factors community and shaped a more inclusive approach to patient safety [ 117 ]. There is an increasing need to understand the diverse organizational factors, ranging from micro-level issues like workflow and communication models to meso and macro-level factors such as change management, politics, and leadership, to better comprehend how these factors impact the implementation and usage of health information technology (HIT) within a setting [ 13 ]. The meso level encompasses system behaviors and user interactions that extend beyond individual users to organizational systems, including the scaling of individual tasks into team-based tasks. In human factors analysis, the macro level refers to the broader organizational and environmental context in which systems and processes operate. This level focuses on large-scale factors that influence the performance, behavior, and interaction of individuals and groups within an organization such as policies and regulations and change management.

### 3.9. A Framework for Investigating and Resolving Technology Implementation Issues

There have been significant advances in health technologies. However, their potential to improve patient health outcomes remains equivocal as discussed by Greenhalgh *et al.* [ 118 ]. Many of the problems include nonadaptation, abandonment, and difficulties in scaling and sustaining innovations. There are a plethora of human factors informed frameworks to account for the barriers and facilitators. Greenhalgh *et al.* argue that technology implementation frameworks have significant limitations. They often assumed a “textbook” condition that oversimplified the complexity and heterogeneity of real-world health issues. Many frameworks failed to account for socioeconomic and demographic disparities in access and use of health technologies. Additionally, few frameworks assessed the overall value proposition, determining whether a new technology is worth introducing from the vantage point of the various stakeholders ( *e.g.* , patients, healthcare institutions, and developers) given the various tradeoffs. The authors sought to create an evidence-based, theory-informed, and practical framework to aid in the design, implementation, and sustainability of health technology programs.

Greenhalgh *et al.* [ 118 ] introduced the NASSS (Nonadoption, Abandonment, Scale-up, Spread, and Sustainability) framework, a comprehensive model developed to predict and evaluate the success of technology-supported health or social care programs by addressing key challenges across seven domains: the condition or illness, the technology, the value proposition, the adopter system, the organization, the wider context, and the interactions over time. NASSS emphasizes the complexity and dynamic interactions of these domains, advocating for adaptive, context-specific approaches rather than rigid models. The framework aims to provide a more nuanced and practical guide for implementing and sustaining health technologies.

A PubMed search using the NASSS keyword yielded 48 results with a wide range of studies and systematic (and scoping) reviews across diverse geographic regions that employed the framework over the past several years. Here, we describe a few noteworthy ones. Winter and Chico [ 119 ] used NASS to identify barriers and facilitators for the implementation of digital twins in cardiovascular medicine. A digital twin is a virtual representation of a complex system, updated with real-time data from its physical counterpart. It is gaining traction in medicine, particularly for improving cardiovascular disease prevention. The study identified key facilitators such as disease prevention in silico and personalized care, and barriers including real-time data exchange, specialist skills, data demand, and privacy concerns.

As discussed, wearable health monitors are increasingly used across a range of health conditions. According to Liverani *et al.* , [ 120 ], they could be employed to strengthen responses to cardiovascular and other non-communicable diseases (NCD) in low- and middle-income countries like Cambodia. Using the NASSS framework, this study identified three potential applications: health promotion, patient monitoring, and NCD risk factor surveys. Challenges included issues related to adopters, national health system organization, infrastructure, regulation, and the technology itself. At this time, wearables are most viable for conducting risk factor surveys in this context. Future use for patient monitoring and management requires careful consideration of feasibility issues and organizational factors.

Patients with recurrent urinary tract infections (rUTI) often lack sufficient knowledge about preventive measures to reduce their risk of future UTIs [ 121 ]. Pat and colleagues developed and assessed the feasibility of myRUTIcoach, an eHealth system for women with recurrent urinary tract infections (rUTI), using the NASSS framework to guide the analysis [ 121 ]. The evaluation showed high patient satisfaction, with 89% recommending the system. Key facilitators for adoption included enhanced self-management skills, while barriers noted by healthcare professionals included integration issues with electronic health records (EHRs). The authors concluded that while myRUTIcoach has the potential to improve rUTI management, further optimization is needed to enhance its effectiveness and integration within healthcare systems [ 121 ].

### 3.10. Systems Engineering Initiative for Patient Safety (SEIPS)

The NASSS framework has been used to good effect in characterizing the challenges involved in developing, implementing, and using health technologies. As described above, patient safety is a similarly multifaceted, complex and heterogenous problem across contexts. The Systems Engineering Initiative for Patient Safety (SEIPS) model, developed by [ 122 ], is based on the macroergonomic work system model by Carayon and Smith [ 123 ]. It features a detailed description of work system elements, making it applicable to healthcare, and incorporates a quality of care model, enhancing its acceptance in the healthcare community [ 124 ]. The SEIPS model explains how work systems impact care processes, such as care pathways, patient journeys, and workflows, and integrates patient outcomes with organizational and employee outcomes, emphasizing worker safety and quality of working life alongside patient safety. The work system in the SEIPS model includes five components: person, organization, technology and tools, tasks, and environment which affords its broad applicability [ 125 ]. It has been widely used by healthcare researchers, professionals, and educators to study various health information technologies ( *e.g.* , electronic health records, smart infusion pumps, and tele-ICU) and to examine patient safety across multiple care settings [ 124 ]. It has a global reach like the NASSS framework. In this section, we consider the results of several recent studies that employ the SEIPS framework published over the last couple of years.

### 3.11. Teamwork and Workload

Boet *et al* ., [ 126 ] employed the SEIPS model to analyze intraoperative teamwork. A teamwork performance across 50 surgical cases. The model provided a deeper understanding of teamwork behaviors and contextual factors, identifying both optimal and suboptimal practices, thereby revealing critical areas for improvement and promoting a comprehensive understanding of teamwork dynamics. The SEIPS framework was employed in a scoping review to examine the effectiveness of environmental cleaning and disinfection in operating rooms [ 127 ]. The review found mixed results regarding the effectiveness of cleaning practices and stressed the importance of considering all work-system elements and the interdependencies within healthcare environments to optimize cleaning and disinfection processes. In another study, the SEIPS model was applied to map the opioid prescribing process in general practices, revealing high variability and the need for robust procedures, role clarity, and workload management to enhance safety and consistency [ 128 ]. By addressing the complexities and risks, the framework can be employed to promote safer, more efficient prescribing practices that reduce medication errors and improve patient outcomes.

Reuland *et al.* , [ 129 ] employed the SEIPs framework to study the effectiveness of pediatric early warning systems (PEWS) for detecting deterioration of patients' condition in a low-resourced hospital in the Philippines. They identified barriers to the adoption and effective use of the system including limited bed capacity, referral delays, patient overflow, insufficient monitoring equipment, and a high patient-to-staff ratio. Occupational fatigue is a characteristic of excessive workload and reflects the limited capacity to meet work demands. It is a problem that is endemic. The SEIPS model was employed by Watterson *et al.* , [ 130 ] to characterize the multidimensional facets of fatigue including mental, emotional, physical, acute, and chronic fatigue. CancelRx is a health IT system designed to improve communication by automatically sending medication discontinuation information from the clinic electronic health record to the community pharmacy dispensing platform [ 131 ]. Employing a similar approach, Watterson *et al.* , (130) found that CancelRx automated the receipt and processing of medication discontinuation messages, streamlining the process but increasing pharmacists' workload and introduced new errors.

### 3.12. Enhancing Infection Control and Protocol Adherence

The COVID-19 pandemic posed extraordinary challenges for hospitals, particularly in infection control and regulatory reporting. Kaufman *et al.* , [ 72 ] studied infection control processes in a small independent hospital that was designated as a COVID-19-only treatment facility. The study underscores the importance of robust infection control practices and the need for improved digital tools to support these efforts, especially in future public health emergencies. Implementing automated reporting and enhancing data interoperability can mitigate the barriers identified and sustain effective infection control practices.

A scoping review by Jimenez and Lewis [ 132 ] explored infection prevention and control (IPC) in medical imaging departments (MIDs) using the SEIPS model to identify key factors influencing IPC practices. The review highlighted the critical role of hand hygiene, revealing common breaches leading to infections, particularly in contrast-enhanced computed tomography (CT). The study underscores the interconnectedness of SEIPS domains—'persons', ‘organization’, ‘tools and technology’, ‘tasks’, and ‘environment’—influencing IPC. It emphasizes the need for a systems approach to understand and address barriers to IPC, advocating for future research to focus on decision-making processes, procedural steps, education, and tools used in MIDs. Geographical variability in IPC practices suggests tailored strategies are necessary for effective infection control.

Lane-Fall *et al.* , [ 133 ] examined factors associated with high fidelity to standardized handoff protocols during operation room-to-intensive care unit transitions using the SEIPS framework. They found that combinations of conditions, such as the presence of an intensive care unit (ICU) provider, high team attention, and a quiet environment, were critical for high fidelity, demonstrating that multiple factors interact to influence successful protocol adherence. The study concluded that multifaceted strategies addressing various contextual factors are necessary for effective handoff protocol implementation, highlighting the SEIPS model's utility in understanding and improving these processes.

Herlihy *et al* ., [ 134 ] describes the development of the Patient Safety Incident Response Framework (PSIRF) using a user-centered and systems-based approach, particularly the SEIPS model, to replace the previous Serious Incident Framework. The process involved extensive stakeholder engagement, testing, and revisions, focusing on defining response processes, considering work system factors, and integrating feedback loops for continuous improvement. While the SEIPS framework provided a valuable structure for designing PSIRF and emphasized the importance of a systems approach, translating it into practical policy posed challenges, including maintaining detailed connections to systems engineering and ensuring extensive collaboration.

### 3.13. SEIPS and Patient-Centered Studies

The SEIPS model has been instrumental in addressing patient safety healthcare institutions. However, it is increasingly used to focus on health-related activities of patients. Negoescu *et al.* , [ 135 ] conducted a scoping review protocol exploring how patients, and their informal carers manage medications in non-formal settings. The study characterizes the work system elements, processes, and outcome. This review emphasizes the importance of a structured, systems-oriented approach to understanding medication management and ensuring patient safety.

Similarly, a study by Ma *et al* ., [ 136 ] focused on inhalation therapy adherence among asthma and chronic obstructive pulmonary disease (COPD) patients employed the SEIPS 2.0 model to identify factors influencing adherence. Through interviews with patients and healthcare providers, the study highlighted the impact of patient abilities, emotional experiences, and the usability of inhalers, as well as the influence of physical environments and cultural beliefs. These insights underscore the need for tailored interventions that address these multifaceted factors to improve adherence and health outcomes in chronic respiratory disease management.

Kirkendall *et al* ., [ 137 ] examined safer Type 1 Diabetes care at home, the SEIPS model was used to identify failures and potential solutions in medication management. The study involved parents of children with T1D and highlighted common errors such as treatment delays and incorrect insulin administration, suggesting interventions like better communication and real-time decision-making tools to support caregivers.

Wust *el al.* , [ 138 ] introduced a novel Patient Journey Mapping (PJM) method that integrates the SEIPS model to provide a comprehensive view of the patient journey through the emergency department (ED). Conducted in an academic health system's ED, the study focused on older adults and used patient-centered observations, clinician feedback, and focus groups to refine the PJM. The SEIPS-based PJM captures the complexities of non-linear healthcare processes and a holistic understanding of the patient journey, which can be used to evaluate and redesign healthcare processes, identify barriers and facilitators, and serve as a communication tool for patients and care partners.

### 3.14. Culture of Patient Safety

Patient safety is predicated on our ability to control or mitigate human error. It is essential to direct efforts not just at the psychological mechanisms behind human error but also at organizational factors that contribute to human failure. There is ample evidence to suggest studies have shown that many major accidents or adverse events in healthcare can be attributed to organizational failures. Safety culture is defined as the set of characteristics and attitudes in organizations and individuals that prioritize safety as an overriding concern. It is widely believed that that safety culture is a relatively stable, multidimensional, holistic construct that is shared by organizational members [ 139 ]. The reason for studying the safety culture of a given organization that by we may be able to identify problems in the attitudes, norms, and practices of the target groups and organizations [ 140 ]. In turn, this knowledge may be used to guide the planning and implementation of intervention programs enabling the organization to develop improved patient safety practices.

### 3.15. Refining the Safety Culture Construct

Surveys are commonly used to gather data from healthcare professionals, providing insights into their perceptions and experiences about patient safety culture. Falcone *et al* ., [ 141 ] explored the factor structure and construct validity of the AHRQ Hospital Survey on Patient Safety Culture (HSOPS) using data collected between 2017 and 2020 from 191,977 hospital nurse respondents across 320 U.S. hospitals. This study employed an exploratory factor analysis to identify six key factors that influence nurses' perceptions of safety culture: communication lead/speak out/resilience, organizational culture and culture of safety–environment, psychological safety–security/protection, psychological safety–support/trust, patient safety, and communication and reporting for patient safety. All factors had moderate to strong associations, indicating the survey's robust factor structure. The authors concluded that emphasizing resilience can promote an environment where errors are viewed as learning opportunities rather than occasions for punitive action, proposing essential elements for fostering an environment of transparent, voluntary error reporting. Lee *et al* ., [ 142 ] also used structural equation modeling to understand how factors like supervisor support and psychological safety influence patient safety outcomes. The study confirmed that psychological safety and management support are critical for effective safety culture. These findings suggest that enhancing these factors can improve patient safety, underscoring the importance of supportive leadership and a psychologically safe environment for healthcare workers.

Duffy *et al.* , [ 143 ] implemented the “One Safe Act” (OSA) tool to highlight proactive safety behaviors in perioperative environments. OSA is a tool for capturing, cataloging, and highlighting proactive safety behaviors and actions used by staff in their daily practice to promote individual and team-based safe patient care. The OSA emphasized the importance of learning within a community of practice. By sharing proactive safety behaviors, staff could build shared knowledge and practices that enhance patient safety. Shifting the focus from what goes wrong to what goes right aligns with the Safety-II framework [ 144 ], which emphasizes resilience and adaptability in healthcare settings.

### 3.16. Global Perspectives on Patient Safety Culture

Kaya *et al* ., [ 145 ] examine the effects of various dimensions of patient safety culture on four key outcomes: self-reported errors, witnessing errors, incident reporting, and patient safety grade. The data were collected using the Turkish version of the Safety Attitudes Questionnaire. When the overall patient safety culture score increased by 1 point; the probability of witnessing an error was 2 times lower, the probability of incident reporting was more than 4 times higher), and the probability of assessing the patient safety grade as excellent was almost 30 times higher. The teamwork climate was negatively related to making errors and witnessing errors.

Lee and Jang [ 146 ] investigated how nurse professionalism, work environment, and communication with healthcare professionals influence patient safety culture in six South Korean tertiary hospitals. A multiple regression model was used to analyze variables influencing patient safety culture. The factors such as nursing foundations for quality care, nurse manager leadership, and supportive nurse-physician relations positively impact patient safety culture. To ensure effective communication in healthcare settings, nurse managers should prioritize shift communication, assess the accuracy of information exchange, and implement various communication channels, such as social media platforms and business messengers, for internal hospital communication. Effective communication, including accuracy, timeliness, and shift communication, is crucial for fostering a positive safety culture. The study emphasized the need for a systematic, organizational approach to enhance patient safety, highlighting the importance of continuous education and strong leadership to support nursing quality and collaboration among healthcare professionals.

In a similar vein, a study of healthcare workers in Riyadh, Saudi Arabia hospitals showed generally low awareness of patient safety culture [ 147 ]. The study highlighted the necessity of training programs to improve understanding and practices related to patient safety and leading to better patient outcomes and a more supportive work environment. A cross-cutting theme is the need to emphasize a non-punitive culture to encourage error reporting and creating a blame-free environment for healthcare professionals. Fekonja *et al.* , [ 148 ] conducted a cross-sectional study of the views of 201 triage nurses from 11 emergency departments in Slovenia regarding patient safety culture. The results revealed that the overall average perception of the patient safety culture among triage nurses in the emergency department was not positive. Significant differences in safety culture perceptions were associated with age, education level, and years of experience, with older, more educated, and more experienced nurses showing more positive perceptions. The study emphasizes the need for systematic improvements in the work environment, leadership, and communication to enhance patient safety culture. It highlights the critical role of effective leadership and the necessity for targeted interventions to foster a robust safety culture, ultimately improving patient safety and care quality in emergency departments.

Watari *et al* ., [ 149 ] explored the perceptions of patient safety culture among medical residents in Japanese university and community hospitals. The study surveyed 5,968 first and second-year residents using the Safety Attitudes Questionnaire (SAQ) adapted to the Japanese healthcare context. Results indicated that residents at community hospitals perceived a significantly better safety culture compared to those at university hospitals. Factors positively associated with better safety culture included more emergency department duties and incident reporting experiences. The study underscores the need for targeted improvements in safety culture at university hospitals, emphasizing the importance of effective communication, supportive environments, and comprehensive incident reporting training. These findings highlight the critical role of organizational support and active engagement in fostering a robust safety culture among medical residents, ultimately aiming to enhance patient safety outcomes across healthcare settings.

Fuseini [ 150 ] evaluated the perceptions of patient safety culture among nurses in emergency and critical care services in a maternal and child health department in Portugal. Using HSOPS survey, the study found that only teamwork within units scored above 75%, indicating strong collaboration among nurses. However, non-punitive responses to errors and open communication were rated the lowest, highlighting significant areas for improvement. The overall average positive score of 49.4% is below the Agency for Healthcare Research and Quality (AHRQ) benchmark of 75%, suggesting that patient safety is not a sufficiently high priority. In keeping with other research, the study emphasized the need for a non-punitive work environment, better communication, and continuous training to improve patient safety culture especially in high-risk areas like maternal and child health.

Curtis *et al* ., [ 151 ] explored how the Australian Medical Assistance Team (AUSMAT) established a strong safety culture at a quarantine facility during the COVID-19 pandemic. The study used a cross-sectional survey to assess perceptions, barriers, and facilitators of infection prevention and control (IPC) among 101 participants. Results showed high agreement on the success of IPC procedures, driven by motivations to protect oneself, family, and the community rather than workplace pressures. Daily training and quality personal protective equipment (PPE) were deemed essential, although environmental factors like heat and fatigue posed challenges. The findings highlight the effectiveness of a multifaceted strategy to develop safety culture in managing high-risk environments and suggest that similar strategies could be applied in other emergency response and quarantine settings.

### 3.17. Burnout and Patient Safety Culture

Vail *et al* ., [ 152 ] examined the relationship between teamwork and burnout among hospital staff at military treatment facilities using data from the 2019 Department of Defense Patient Safety Culture Survey. The study found that about one-third reported experiencing burnout. High burnout rates were particularly noted in work areas such as pharmacy, labor and delivery, and emergency departments, and among staff positions like pharmacists, assistants, technicians, and nurses. The study revealed that effective teamwork within and across units significantly reduced the odds of burnout, with teamwork within units showing the strongest protective effect. Dimensions such as proper staffing, management support for patient safety, and communication openness were also associated with lower burnout levels. The findings underscore the importance of fostering a positive patient safety culture through improved teamwork and organizational support to mitigate burnout.

Sousa *et al.* , [ 153 ] investigated the association between occupational burnout and patient safety culture among healthcare workers in 18 Primary Health Care Units in Northeast Brazil. The study surveyed 78 healthcare workers, revealing that 64.1% had a reduced risk of burnout, while 11.5% had a high risk. Weakened dimensions of safety culture, such as work pressure, leadership support, and overall ratings on quality and patient safety, were linked to a higher risk of burnout. The study underscores the importance of fostering a positive safety culture to mitigate burnout, highlighting that effective interventions in safety culture can prevent the detrimental effects of burnout on patient care. The findings suggest that enhancing leadership support, improving work conditions, and promoting open communication are crucial strategies for reducing burnout and improving patient safety in primary healthcare settings.

Kim *et al.* , [ 154 ] investigated the associations between patient safety culture, workplace violence (mostly acts committed by patients), and burnout among healthcare workers. The findings indicate that nurse burnout levels are significantly higher than those of allied health professionals, with both nurse and physician burnout exceeding the overall average. Nurses exhibit significantly higher burnout levels compared to allied health professionals, with both nurses and physicians experiencing higher-than-average burnout rates. Positive perceptions of teamwork across units and effective handoffs and transitions are associated with lower levels of burnout. These factors also correlate with fewer instances of physical and verbal violence from patients. System-level improvements, such as better staffing, management support, and open communication channels, are essential for creating a safer and more supportive work environment.

## 4. Conclusions

Human factors and organizational systems research is a vibrant and dynamic field that continues to expand in scope and global reach. In this paper, we examined a range of phenomena from micro-level issues affecting individual technology users to meso-level issues impacting healthcare teams and institutions. This has included continued work in areas such as participatory design of systems, improved user requirements modelling and specification, improved methods for ensuring healthcare system safety, and integration of new applications such as AI tools and systems into healthcare in ways that do not interfere with workflow and that can augment and extend healthcare work. In addition, in line with current trends, the initial sections of the paper explored technologies involved in clinical practice, patient-facing technologies, and communication-facilitating technologies such as telehealth. The latter sections addressed meso and macro issues involving larger entities, specifically focusing on studies employing approaches such as the SEIPS model to address practical workflow matters. Additionally, we examined patient-safety culture research, an area that had been neglected until recent years. There have been global efforts to better understand and improve safety culture, though it remains a challenging endeavor. We surveyed a broad range of human factors and organizational research, noting exceptional growth in knowledge in recent years.

However, challenges remain including standardizing approaches and instruments and improving scholarly communication. Knowledge translation, involving moving from research studies to practical application of new methods for improving user interactions with systems such as EHRs has remained a challenge. This has been particularly the case when considering commercial vendor based systems, where application of research findings for improving system usability remains challenging. This has also included a continued need for better understanding of human factors issues by healthcare decision makers and management ( *e.g.* , chief information officers), as the success of healthcare systems will depend on their effectiveness, usability and usefulness from the user perspective [ 3 ]. Other challenges remain around developing design of healthcare applications that are truly user-centric and that effectively support user needs in the varied context of use in healthcare. To address these challenges increased focus on and application of practical models, theories and frameworks will be needed to guide design and evaluation of healthcare IT from a human factors perspective. This review has identified a number of such approaches, ranging from cognitive models, distributed cognition, socio-technical approaches as well as team-based models and frameworks for describing human-computer interaction and communication.

In summary, it is becoming increasingly recognized that the success of health information system, and more generally health informatics as a field, will depend on continued work in advancing human factors research as well as practical application of research methods and findings. Over the past two years a wide range of approaches to design and evaluation of healthcare IT have appeared, as described in this paper. It is expected that many of these trends will only be accelerated in coming years, as the scope, diversity and range of healthcare systems, technology rapidly expands. The importance of human factors and organizational issues is underscored by the continued challenges reported with the adoption of health information technology and systems. Indeed, the success of health informatics initiatives will ultimately depend on an improved understanding of human factors and organizational issues in health informatics.
